# A Comparative Interrupted Times Series on the Health Impact of Probiotic Yogurt Consumption Among School Children From Three to Six Years Old in Southwest Uganda

**DOI:** 10.3389/fnut.2020.574792

**Published:** 2020-12-09

**Authors:** Nieke Westerik, Arinda Nelson, Alex Paul Wacoo, Wilbert Sybesma, Remco Kort

**Affiliations:** ^1^Yoba for Life Foundation, Amsterdam, Netherlands; ^2^Department of Molecular Cell Biology, Vrije Universiteit Amsterdam, Amsterdam, Netherlands; ^3^Department of Food Technology and Nutrition, School of Food Technology Nutrition and Bioengineering, College of Agricultural and Environmental Sciences, Makerere University, Kampala, Uganda

**Keywords:** lactobacillus rhamnosus yoba 2012, yogurt, milk, respiratory tract infection, common cold, tinea capitis, school feeding program, probiotics

## Abstract

**Introduction:** Following a school milk feeding program in Southwest Uganda, we initiated a probiotic yogurt school feeding program in the same region in 2018. In order to investigate the potential health benefits from probiotic yogurt we conducted an observational study, where we compared the effect of the consumption of locally produced probiotic yogurt containing *Lactobacillus rhamnosus* yoba 2012 to milk in pre-primary schoolchildren from different schools on the occurrence of respiratory tract infections (common cold) and skin infections (e.g., tinea capitis).

**Method:** A comparative interrupted time series over a period of 3 weeks of baseline followed by 9 weeks of 100 ml of probiotic yogurt or milk consumption for 5 days per week. In total 584 children attending five different schools were followed during consumption of probiotic yogurt and 532 children attending five other schools during consumption of milk. Incidences of respiratory tract infection symptoms and skin infection symptoms, changes in anthropometric indicators and absenteeism were recorded.

**Results:** Over the course of the study period the incidence rate for common cold symptoms decreased faster in the yogurt group than in the milk group (*p* = 0.09) resulting in a final RR of 0.85 (95% CI: 0.5–1.4) at the end of the observational period. The incidence rate of skin infection related symptoms also reduced faster in the yogurt group compared to the milk group (*p* < 0.0001) resulting in a relative risk factor (RR) of 0.6 (CI: 0.4–0.9) at the end of the observational period. Anthropometric indicators and level of absenteeism did not show significant differences between yogurt and milk.

**Conclusion:** Notwithstanding the observed positive trend and effect of probiotic yogurt on the incidences of common cold and skin infections, respectively, we consider the results of this comparative interrupted time series inconclusive due to differences in the recorded health parameters between the probiotic yogurt and milk control groups at base line, and fluctuations over the course of the intervention period. An improved study design, with more uniform study groups, a longer intervention period and a third control group without yogurt or milk is required to draw definitive conclusions.

## Introduction

### Disease Incidences

Children in developing countries such as Uganda are at high risk of morbidity due to common childhood diseases. The Ugandan National Demographic and Health Survey indicated that in the Southwestern region of the country, 14% of the children below 5 years of age had suffered from diarrhea, and 11% from Respiratory Tract Infections (RTIs) (1). *Streptococcus pneumonia*-related RTIs together with other lower respiratory tract infections occur in 3% of all children younger than 5 years old in Africa (2) and are the leading cause of child mortality in Uganda. They are responsible for 23% of the deaths of children between 1 and 59 months of age (3). Tinea capitis, a fungal infection, usually caused by *Trichophyton* or *Microsporum* species on the scalp, is a common skin condition among children 2–11 years (4). Tinea capitis is found worldwide, but is more common among children living in crowded households and under poor sanitary conditions, as is commonly found in developing countries such as Uganda (4, 5). National statistics on the incidence of this disease in Uganda are not available. Finally, in the Ankole Region in Southwest Uganda, 29% of the children below 5 years old have been found to be stunted, and 2% is wasted (6). Aflatoxins, carcinogenic substances produced by molds in poorly stored foods, have been proposed as a precursor for stunting (7). Aflatoxins are found in alarmingly high levels in commonly consumed foods in Uganda, most especially in ground nuts and maize (8).

### Milk School Feeding Program

The World Health Organization (WHO) recommends the intake of 0.66 g protein/kg body weight per day, as protein is the best source of amino-acids (9). Like all developing countries, the diet in Uganda is starch-dominated, and hence many people do not meet the daily recommended intake of protein (10). Milk is an excellent source of protein with high bioavailability (11). Facing the paradox of poor child growth indicators vs. the high production of milk in the southwestern region, SNV (The Netherlands Development Organization) designed a program to promote the consumption of milk in schools as part of a larger developmental project in the dairy sector in this region, called The Inclusive Dairy Enterprise Project (TIDE). Under this program, primary schools in seven districts in Southwest Uganda that are part of the Ankole sub-region (Kiruhura, Lyantonde, Bushenyi, Sheema, Isingiro, Ntungamo, and Mbarara) have been participating. School leaders and parents have been sensitized about the health benefits of regular milk consumption for child development and general well-being, and encouraged to pay an additional school fee for the milk. For ~5.5 USD, a child receives 100 ml of milk for 5 days per week during a school term of 12 weeks. Raw milk is delivered and boiled at the school premises as a component of maize porridge, after which it is consumed by the children in the form of a hot beverage. After 4 years of implementation, ~300,000 primary and pre-primary school children have been enrolled in the program.

### Yogurt School Feeding Program

Following the success of the TIDE school milk program, SNV and the Yoba for Life foundation designed a similar program including locally produced probiotic yogurt instead of milk. The rationale for shifting from milk to probiotic yogurt comes from the assumption that the probiotic bacteria, especially *Lactobacillus rhamnosus* yoba 2012, the generic version of *L. rhamnosus* GG, could boost the immunity and alleviate infections and diseases that are frequently occurring in young children, including diarrhea, common cold, allergies, skin conditions and growth retardation.

### The Probiotic Bacterium *Lactobacillus rhamnosus* GG

The bacterium *L. rhamnosus* GG is the world's best documented probiotic with a number of proven health benefits (12), including the prevention and reduction of diarrhea (13), common cold (14), allergies and skin conditions (15). The probiotic strain used in the locally produced probiotic yogurt in Uganda is a generic variant of *L. rhamnosus* GG, called *Lactobacillus rhamnosus* yoba 2012 (16). Over 30 studies have been conducted on the effect of probiotics on children attending day care centers (17, 18), the majority of them looking at the incidence and duration of gastrointestinal infections and respiratory tract infections (RTIs). No adverse effects of the consumption of *L. rhamnosus* GG have been reported in children to date. An overview of the studies that use *L. rhamnosus* species is presented in Supplementary Table 1. In the current study, we specifically look at the incidence of common cold and skin infections. Common cold is the most common form of respiratory tract infection.

With regard to skin infections, a number of studies have been conducted on the incidence of tinea capitis among primary school children in various African countries. For example, Ayaya et al. (5) found an incidence of 33% among children attending a primary school in Kenya, Chepchirchir et al. (19) found 11% among eight primary schools in urban slums in Kenya, and Ngwogu and Otokunefor (20) found an incidence of 20% among children attending primary schools in Nigeria. Two *in vitro* studies showed a strong inhibitory effect of lactic acid bacteria species on the growth of the fungal species *Microsporum* and *Trichophyton*, which are responsible for tinea capitis (21, 22). The mechanism of action presumably comes from the production of anti-fungal compounds. To date no studies have been performed on effects of probiotic intake on tinea capitis in humans resulting from a presumed immune modulatory mechanism.

In spite of the large body of evidence showing the ability of *L. rhamnosus* GG to prevent and reduce the above-mentioned conditions, the efficacy of *Lactobacillus rhamnosus* has not been studied in the population of Ugandan children. Yet in Uganda, there is an established and sustainable supply of *Lactobacillus rhamnosus* yoba 2012 through the local production of probiotic yogurt throughout the country by the use of the “yoba” starter culture (23, 24), as well as through the program to promote its consumption among children in pre-primary schools. These practices prompted us to study the health benefits of the probiotic *L. rhamnosus* for this specific population group.

Experimental proof for the efficacy of *L. rhamnosus* GG has been described in many studies, mostly in the form of probiotic supplements. However, this is the first time that we evaluate health effects of the consumption of *L. rhamnosus* yoba 2012 in children consuming locally produced probiotic yogurt containing *L. rhamnosus* yoba 2012 and *S. thermophilus* C106. Only children from whom the parents had agreed to pay for yogurt or milk consumption as ongoing practice participated in this study.

### Local Production of Probiotic Yogurt

Since 2012 the local production of probiotic yogurt has been promoted in Uganda, resulting in a production network of more than 130 small scale yogurt production units (24). The yogurt program is similar to the school milk program and has been implemented in the same area, but targets specifically pre-primary schools. The cost of taking 100 ml of yogurt for 5 days per week is 6.50 USD per school term. The program intends to simultaneously support the health of young children, as well as creating a market for the locally produced yogurt, thereby facilitating women empowerment, increasing employment opportunities and raising household incomes (24, 25). Since the beginning of the program in 2018, over 20,000 pre-primary children have been enrolled in the probiotic yogurt school feeding program.

## Methods

### Subjects and Design

This study followed a comparative interrupted time series design (CITS), carried out over a period of 12 weeks, and included 1,116 children aged 3 to 6 years old. The study compared health related parameters of children consuming 100 ml of milk per day to children consuming 100 ml of probiotic yogurt containing *Lactobacillus rhamnosus* yoba 2012 and *Streptococcus thermophilus* C106 per day, for 5 days per week during 3 weeks of baseline and 9 weeks of dairy consumption. Five schools participated in the milk group (Faith Memorial Nursery and Primary School Bushenyi, Queen and King Nursery and Primary School Itojo Ntungamo, St. Eliza Excell Nursery and Primary School Isingiro, Jireh Junior School Ishaka Bushenyi, St. Francis Nursery and Primary School Lyantonde and Primary School Isingiro Itojo, with 171,132, 85, 74, and 70 children, respectively, total *N* = 532) and five schools participated in the yogurt group (Blue Sight Primary School Kabwohe Sheema, Hanny Nursery and Primary School Isingiro, Mbarara Progressive Nursery and Primary School, BDA Nursery and Primary School Ishaka Bushenyi, Itojo Nursery and Primary School Itojo Ntungamo, with 177, 139, 115, 77, and 76 children, respectively, total *N* = 584).

Selection of schools was based on enrolment of the school in the yogurt or milk program as promoted by SNV and the Yoba for Life Foundation. This implied that the parents agreed to pay for the milk or yogurt consumption of their children, not only for the duration of the study, but as an ongoing practice. This selection criterion ensured that children will profit from long-term benefits from yogurt or milk consumption. Before participation in the participating study, written consent was obtained from parents or caregivers of all participating children.

### Intake of Dairy Products

Milk was supplied by local farmers or dairy cooperatives who were identified by SNV/TIDE in collaboration with the school. The milk was delivered to the schools as fresh milk in aluminum milk cans, and tested with milk quality tests, as described previously (26). The milk was boiled at the school premises together with water and maize flour, into a maize porridge. Each child in the study consumed 400 ml maize porridge per day, which contained 100 ml milk.

The yogurt for the five schools was produced by five local producers (Nunu probiotic yogurt, Mbarara; Tiana Foods, Mbarara; Blessed Choice, Ntungamo; Rwembogo, Isingiro; K-yoba, Sheema). The yogurt was made using a starter culture containing *Lactobacillus rhamnosus* yoba 2012 and *Streptococcus thermophilus* C106 (26) and contained 5% (w/v) sugar and 0.1% (w/v) artificial flavor (strawberry or vanilla). The yogurt was packed in small, specifically designed polythene bags of 100 ml (Supplementary Material Picture 1), as is a common practice in Uganda. The yogurt was delivered at the schools during weekdays at the agreed delivery time. The drinking yogurt was consumed by the children with a straw. Waste bins were provided to the schools for plastic waste collection. On a daily basis, the quality of the yogurt was monitored by organoleptic tests (taste and appearance) before being served to the children. In collaboration with the local authorities (Dairy Development Authority), samples of the five producers were taken twice during the study in order to test for the presence of pathogenic bacteria.

In accordance with the cell count experiments as described previously (23, 26), a daily serving size of 100 ml of the probiotic yogurt contains ~5 × 10^9^ CFU *L. rhamnosus* yoba 2012 and 1 × 10^11^ CFU *S. thermophilus* C106 per day.

### Parent Questionnaires

Three times during the study, in week 1 and 2, 5 and 6 and 11 and 12 a questionnaire (Supplementary Text 1) was conducted among the parents of the participating children. The objectives of this questionnaire were to collect data about (1) the socio-demographic characteristics of the children that might affect the study outcomes, (2) the diet of the child, specifically with regards to the intake of dairy products and fermented foods, (3) the health status of the child for cross checking and complementing the data that were collected by the nurses (see below).

Dietary information was evaluated and quantified using the Dietary Diversity Score (DDS) tool and assessed the variety of foods that a household accesses and consumes on an average day, as reported by Swindale and Bilinsky (27). Food diversity was scored between 0 and 12, based on a recall of food eaten in the last 24 h from a maximum of 12 different food groups.

### Absenteeism

Teachers in every school kept daily track of child absenteeism by using specifically designed attendance lists.

### Monitoring of Common Cold Symptoms and Skin Conditions

Five nurses were engaged in the study and visited the schools 5 days per week to monitor the incidence of skin infections and common cold related symptoms among the children. The school nurses observed every child individually, thereby focusing on detecting signs of common cold, including cough, runny nose, blocked nose, sore throat, fever, headache, malaise, loss of appetite, as well as skin conditions, including any type of irregularity, infection, wound or rash on the skin. In case of absenteeism, the teacher was asked to try to reach the parent of the absent child by phone, to inquire for the reason for absence, which was subsequently recorded by the nurse.

All nurses were equipped with tablets that contained a software application (app) (Kenga Mobile, OMNI-Tech Ltd, 2018) that was specifically developed for this study (downloadable from Google Play Store). The app facilitated the reporting on skin infections and common cold related symptoms for each individual child (see Supplementary Text 1). For skin infections, the type and the part of the body that was affected, were specified, and a picture of the affected body part was taken as part of the app questionnaire. For common cold, the specific symptoms were indicated via the app. No therapeutic actions were supposed to be taken by the nurses, except in case of emergencies.

### Anthropometric Indicators

**Figure 1:**
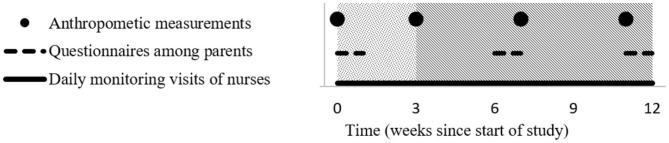
Timeline indicating the moments of different data collection. Dotted area = baseline. Diagonal striped area = milk/yogurt consumption period.

Anthropometric data were taken four times during the study (Figure 1), including weight, as measured on a digital weighing scale (Casa CEGS01, South-Africa) and height as measured with a height rod with stand (Fazzini S225, Italy). All equipment was calibrated, and the nurses had been collectively trained on its use and were individually coached and monitored during their activities. Training of the nurses had been performed according to the WHO guidelines for standardized measurement procedures (28).

### Data Collection and Analysis

All data was collected through a specifically designed mobile software application, mentioned before, from where it was uploaded onto a protected online platform with access only for the researchers. All children were registered in the app. The app contained three different “forms” (see Supplementary Text 1), which were linked to each child: 1. a form for parent questionnaires, 2. a form for reporting incidences of diseases, and 3. a form to report anthropometric measures. From the online data collection platform, data was exported to Excel for further analysis.

### Propensity Score Weighting

For analysis of the incidence of common cold, skin infections and absenteeism, the percentage of the total study group that was sick, was considered as the unit of analysis. Propensity score weighting was used to correct for differences in baseline characteristics of the children. Propensity scores were calculated based on socio-demographic characteristics as obtained through the parents' interviews at baseline. Weighting was done based on both the standardized mortality ratio weighted (SMR) method, as described by Kurth et al. (29). The incidence of common cold, skin infections and absenteeism were treated as individual interrupted time series, and analyzed according to the segmented regression methods, as described by Wagner et al. (30) and Linden and Adams (31). Model fitted trend lines were obtained through (1) three median smoothing (datapoint replaced by median of the data point, the preceding datapoint and the following datapoint, (2) hanning (0.25 xprevious data point + 0.5 xdata point + 0.25 xfollowing data point), and (3) skip mean (average of the previous and the following data point). All hypothesis tests used a significance level (α) of 0.05. All analyses were done using Microsoft Office Excel 2019.

### Analysis of Anthropometrics

Anthropometric records were evaluated with software provided by the Word Health Organization (WHO Anthro version 3.2.2 and WHO AnthroPlus version 1.0.4) (32, 33). With this software package, measures of weight and height were assessed in reference to the WHO standard growth curves, and expressed as Z-scores, which are the number of standard deviations away from the standard value as obtained from the standard growth curves (34, 35).

## Results

### Socio-Demographic Characteristics and Propensity Score

Socio-demographic characteristics of the children in the yogurt and milk group were very similar in the average age of the household head, the composition of the household, sanitary conditions, and methods of preparing drinking water (Table 1). However, differences were pronounced in the proportion of female-headed households (widows or single mothers), level of education of the parents, source of water (e.g., piped water or well) and school fees paid. More specifically, on average children in the yogurt group belonged to higher income families compared to children in the milk group. Hence, a propensity score that was calculated based on socio-demographic characteristics as obtained through the parents' interviews at baseline, was assigned to the study subjects. Next, propensity score weighting was used to correct for differences in baseline characteristics of the children. As a result, the similarity of socio-demographic characteristics in the control and yogurt group improved notably, as is shown in Table 1.

**Table 1:** Household characteristics of the yogurt group (*n* = 577) and milk group (*n* = 492), collected during the baseline questionnaire in the first 2 weeks of the study.

**Category**		**Original**		**SMR-weighted**	
		**Milk**	**Yogurt**	**Milk**	**Yogurt**
Gender	Boys	51%	52%	55%	52%
	Girls	49%	48%	45%	48%
Average age child		4.84	4.79	5.14	4.79
Household	Male headed	80%	89%	87%	89%
	Female headed	20%	11%	13%	11%
Average age household head		36.37	36.60	38.15	36.60
People in household		5.32	5.53	5.66	5.53
Females ≤ 5 years		0.85	0.85	0.89	0.85
Males ≤ 5 years		0.84	0.87	0.95	0.87
Females 6–13 years		0.50	0.57	0.45	0.57
Males 6–13 years		0.60	0.66	0.64	0.66
Females 14–59 years		1.32	1.26	1.40	1.26
Males 14–59 years		1.06	1.09	1.14	1.09
Females ≥ 60 years		0.09	0.11	0.10	0.11
Males ≥ 60 years		0.05	0.13	0.08	0.13
Education level head	No formal education	4%	2%	2%	2%
	Primary	31%	18%	24%	18%
	Secondary	29%	33%	27%	33%
	Tertiary	37%	46%	47%	46%
	Toilet	98%	98%	98%	98%
Sanitary condition	No toilet	2%	2%	2%	2%
	Protected well or spring	12%	5%	8%	5%
Source of water	Borehole	5%	3%	2%	3%
	Open spring or well	17%	9%	9%	9%
	Surface water	1%	0%	0%	0%
	Rain water	2%	3%	1%	3%
	Piped water	63%	79%	81%	79%
Drinking water	Boil water	99%	100%	98%	100%
	Let it stand and settle	1%	0%	1%	0%
Food taken in the last 24 h	Fermented cereal (bushera)–baseline	26%	8%	9%	8%
	Fermented cereal (bushera)–midline	12%	14%		
	Fermented cereal (bushera)–end line	10%	11%		
	Milk	72%	58%	68%	58%
	Fermented milk	3%	4%	3%	4%
School fees		127	196	127	196

### Dietary Indicators

Dietary diversity scores (DDS) calculated at baseline, midline and end line for the yogurt group were 6.4, 6.5, 6.8, respectively, and for the milk group 6.2, 6.5, 6.5, respectively. The DDS in the yogurt group was slightly higher, possibly as a result of a relatively higher wealth status, as mentioned before. On an average day before the study started, 58% of the children in the yogurt group and 72% of the children in the milk group would consume dairy products (Table 1). As a result of the yogurt or milk consumption, this increased to 88% in the yogurt and 91% in the milk group (during weekdays every child consumed a milk product, but on Saturday and Sunday this depended on household choices). The increased consumption of milk products explains for a large part the slight increase of DDS in midline and end line as compared to baseline.

At household level, on an average day during the study, 11% of the children in the yogurt group and 16% of the children in the milk group consumed a fermented cereal porridge called bushera (36). In addition, on an average day during baseline, 4% of the children in the yogurt group and 3% of the children in the milk group consumed fermented milk products (locally fermented milk or regular yogurt).

### Evaluation of Skin Conditions and Common Cold Symptoms

Data from skin condition observation and common cold collected during the 5 weeks of study by the locally employed nurses at the ten different schools, are presented in Supplementary Figures 1A–F.

### Skin Infections

During the 12 weeks of the study, 30% of the children in the yogurt group and 35% of the children in the milk group were found to have a pathological skin condition at some point in time. The most common identified skin conditions occurred on the scalp, i.e., tinea capitis (fungal infection) and folliculitis (bacterial infection of hair follicles). Usually, the children suffered from a combination of both conditions. These two scalp conditions together accounted for 89 and 90% of all the reported skin conditions in the yogurt and milk group, respectively.

**Figure 2:**
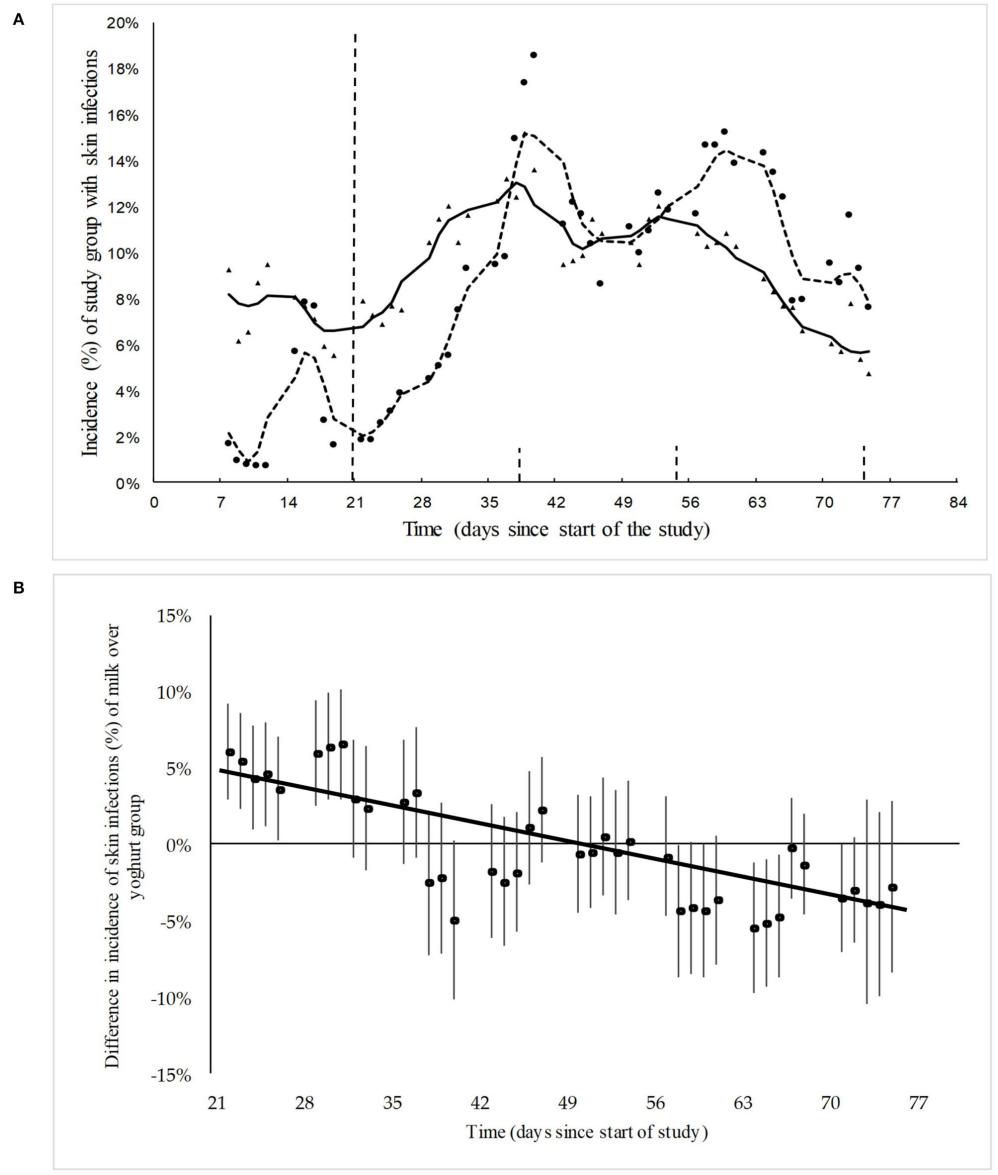
**(A)**The actual and model-fitted SMR-weighted incidence of skin infections in the yogurt group (*n* = 507) group and milk group (*n* = 287) between day 7 and day 73, expressed as a percentage of the total children under observation. Δ = data points yogurt group; solid line = trend yogurt group; • Data points milk group; dotted line = trend milk group. The baseline is from day 1 to day 21 and the yogurt/milk consumption period is from day 22 to day 84. **(B)** The actual and model-fitted difference and the 95% confidence intervals in the relative incidence of skin infections in the yogurt group over the milk group as recorded after baseline period.

During the 3 weeks of baseline measurement, the incidence of skin conditions was found to be higher in the yogurt group compared to the milk group, with averages of 7 and 3%, respectively (Figure 2A). In the subsequent 3 weeks, which were the first 3 weeks after the start of intake of yogurt or milk, the incidence of skin conditions rose substantially in both groups. However, this rise is stronger in the milk schools compared to the yogurt schools group. Furthermore, after a peak at 6 weeks, the incidence of skin conditions in the yogurt group decreased, while the incidence in the milk group continued to fluctuate with an average decrease over the remaining 4 weeks of (Figure 2A).

The difference in the relative incidence of skin infection symptoms between the yogurt group and the milk group, and the 95% confidence intervals, are shown in Figure 2B. Linear regression is used to analyse trends, see Supplementary Table 2. After the start of yogurt or milk consumption the incidence of skin infections showed a significant relative decrease over time (*P*-value trendline < 0.0001) in the yogurt group compared to the milk group. This trendline results in a final relative risk (RR) of 0.6 (CI: 0.4–0.9) for the incidence of skin infections after 8 weeks of probiotic yogurt consumption compared to milk consumption. However, as different levels of skin infections were observed in seven out of the ten measurement points during the baseline period for the two groups, care should be taken when interpreting these results.

For a more detailed evaluation of the data, the measurement points during consumption period of yogurt or milk were split-up into three time periods of 13 measurement points, each representing 13 weekdays, as shown by the gray vertical lines in Figure 2A. The average number of days with observed skin infection symptoms per child for each of these time periods is shown in Table 2. In the first time period, incidence and average duration of skin infections were still increasing, probably as a result of cross-infection in the classrooms. However, the relative increase in the yogurt group during this time period was less compared to the milk group. In the second time period, the increase in infections is halted in both groups and in the third time period the average duration of observed skin infections decreased with 26% in the yogurt group compared to a much smaller decrease of 6% in the milk group. These findings are further supported by the analysis of the number of children without reported skin infection symptoms in the same periods of 13 days (Table 2). While in the second time period an increase in the number of children with skin infection symptoms was observed, during the last period the number of children without symptoms increased with 5% in the milk group and 13% in the yogurt group. The observed amelioration suggests a potential beneficial effect of probiotics, which increases over time.

**Table 2:** Comparative analysis of parameters for skin infection and common cold related symptoms per time period of 13 days of measurements during milk and yogurt consumption (a total period of 39 days of measurements in a period of 8 weeks). Note that a negative value means a reduction.

**Parameter**	**Period 1**	**Period 2**	**Period 3**	**Difference**		**Relative difference**		***P*-value**
				**2-1**	**3-2**	**2-1**	**3-2**	
Average number of days of skin infection per child (milk group)	0.75	1.42	1.34	0.67	0.08	89%	−6%	0.30
Average number of days of skin infection per child (yogurt group)	1.34	1.47	1.08	0.13	0.38	9%	−26%	0.0004
Number of children without skin infection (milk group)	222 (77%)	203 (71%)	214 (75%)	−19	11	−9%	5%	0.30
Number of children without skin infection (yogurt group)	402 (79%)	382 (75%)	430 (85%)	−20	48	−5%	13%	0.0002
Average number of days of common cold per child (milk group)	1.16	0.82	0.82	0.34	0.00	−29%	0%	0.71
Average number of days of common cold per child (yogurt group)	1.26	1.08	0.75	0.18	0.33	−14%	−31%	0.097
Number of children without common cold (milk group)	332 (62%)	383 (72%)	379 (71%)	51	−4	15%	−1%	0.79
Number of children without common cold (yogurt group)	395 (68%)	424 (73%)	438 (75%)	29	14	17%	3%	0.35

*Group sizes for skin infections measurements included n = 507 for the yogurt group, and n = 287 for the milk group, and for common cold measurements n = 584 for the yogurt group, and n = 532 for the milk group*.

### Common Cold

**Figure 3:**
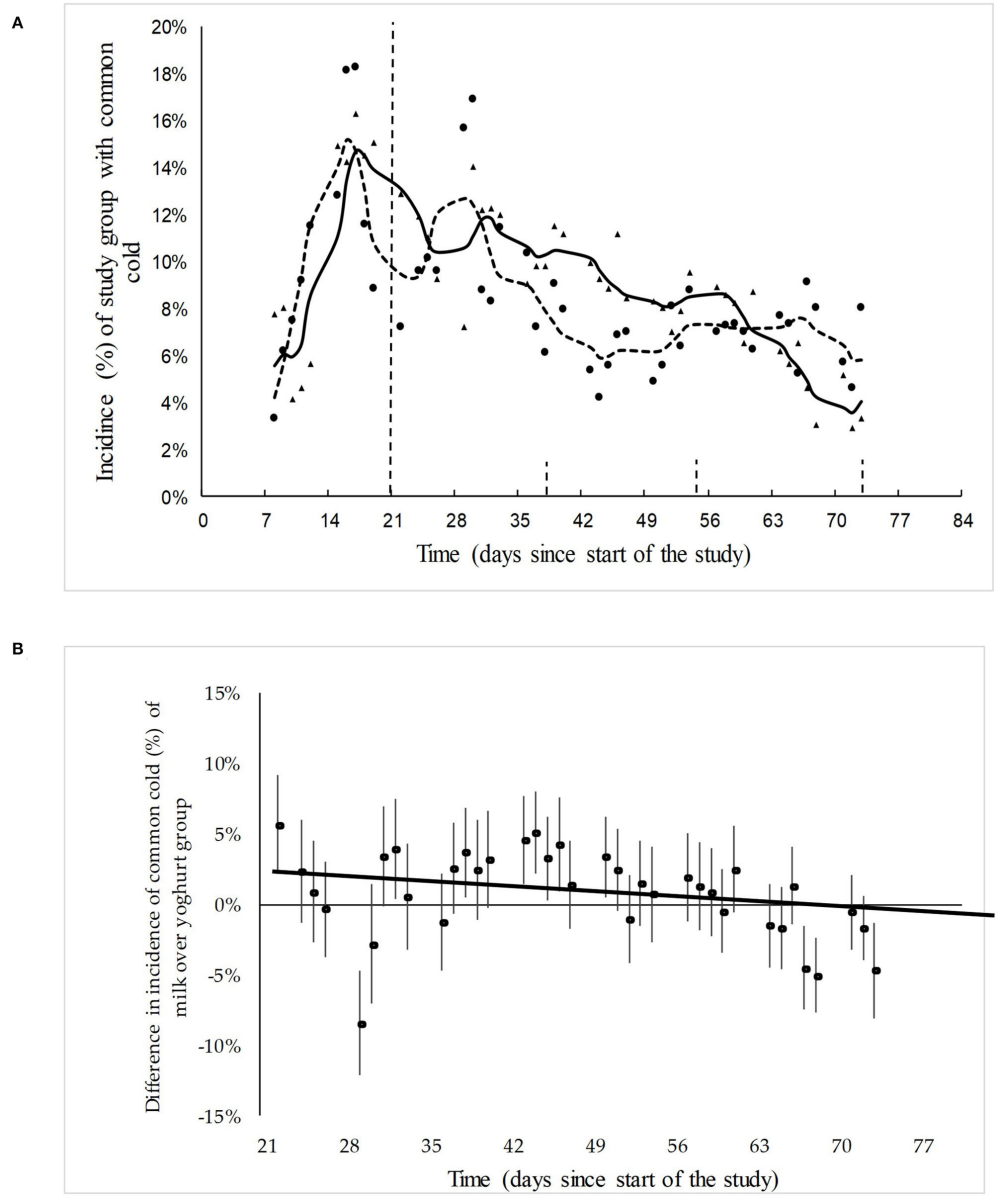
**(A)** The actual and model-fitted SMR-weighted incidence of skin infections in the yogurt group (*n* = 584) group and milk group (*n* = 532) between day 7 and day 73, expressed as a percentage of the total children under observation. Δ = data points yogurt group; solid line = trend yogurt group; • Data points milk group; dotted line = trend milk group. The baseline is from day 1 to day 21 and the yogurt/milk consumption period is from day 22 to day 84. **(B)** The actual and model-fitted difference and the 95% confidence intervals in the relative incidence of common cold in the yogurt group over the milk group during the dairy consumption period.

The incidence of the common cold started to rise immediately when the children went back to school. As soon as the yogurt or milk consumption started there was a trend of decreasing incidence of common cold in the yogurt group as well as in the milk group. However, this trend was slightly stronger in the yogurt group (Figure 3A).

The difference and the 95% confidence intervals in the relative incidence of common cold in the yogurt group over the milk group is shown in Figure 3B. Linear regression was used to analyse trends, see Supplementary Table 2. Following the base line period, there is a slight decrease over time in the number of children suffering from common cold in the yogurt group compared to the milk group (*P*-value trend line = 0.09). This end line results in a final RR of 0.85 (CI: 0.5–.4) for the incidence of common cold after 8 weeks of probiotic yogurt consumption.

Also for this study outcome, the measurement points during consumption of yogurt or milk were split-up up into three time periods of 13 measurement points (representing 13 week days), as indicated by the gray vertical lines in Figure 3A. The average number of days with common cold symptoms per child for each time period is shown in Table 2. Throughout the first and the second time period the incidence and duration of the common cold decreased for both groups. In the last time period, this increase leveled off for the milk group, while it continued to decrease with 31% for the yogurt group. These observations are in agreement with the number of children that have not been reported with common cold symptoms during the three time periods (Table 2). More specifically, in the second time period we could still observe an increase in the number of children with common cold symptoms in both groups. However, during the last time period this changed to a small increase (3%) in the number of healthy children in the yogurt group, while the number of children without symptoms reported for the milk group remained around the same level (−1%).

In-depth analysis of the course of different symptoms of underlying common cold shows that the development of rhinitis is similar to the general course of common cold, while for cough we see a sharp drop in symptoms in the yogurt group as soon as the consumption started. It should be noted that this observed reduction in the cough symptoms was dominated by findings of only one of the five yogurt consumption schools (Supplementary Figures 2A,B).

### Absenteeism

The average absenteeism during week two and three of the baseline period was 3.4% (SD 0.86%) in the yogurt group, and 3.3% (SD 1.1%) in the milk group (Note: the first week was not considered representative as many children had not yet returned after holidays). The average absenteeism during the yogurt or milk consumption period dropped to 2.7% (SD 1.2%) in the yogurt group and 2.3% (SD 0.80%) in the milk group.

### Anthropometric Indicators

The average age, weight and height of the children in both groups at the four measurement points have been indicated at Supplementary Table 3. This table also shows the SMR-weighted average scores for the height-for-age (HAZ), weight-for-age (WAZ) and Body Mass Index-for-age (BAZ) at the four different measurement points during the study. Children in both groups have average lengths and weights, although the average HAZ and WAZ values are slightly below, and BAZ values are slightly above the WHO standards. The children in the yogurt group were slightly shorter for their age compared to the children in the milk group. In this study we found an overall rate for stunting and wasting of 9 and 3%, respectively. Whereas, the rate of stunting is far below the 29% that the Uganda Bureau of Statistics found in the Demographic and Health survey of 2016 (6), the rate of wasting is similar to what has been reported in the survey.

From the results of the current study it can be concluded that the consumption of either 100 ml milk or 100 ml probiotic yogurt per day for 5 days per week, does not have a measurable effect on the growth parameters of children within the relatively short period of 8 to 9 weeks (*P*-value WAZ and BAZ end-line difference yogurt and milk are 0.34 and 0.57, respectively). However, it is remarkable that in both groups the growth parameter scores decreased over the study period for yogurt and milk (Supplementary Tables 3, 4), suggesting that the during the school term the average energy expenditure was higher than the average energy intake.

## Discussion

### Strengths of This Study

The comparative interrupted time series (CITS) was conducted as part of an ongoing developmental program that introduced milk and yogurt in schools, as outlined in the introduction. This is the first time that effects of the consumption of probiotic yogurt containing *L. rhamnosus* yoba 2012 are studied in children attending pre-primary schools on the African continent. Only children were recruited in case the parents agreed to pay for yogurt or milk consumption as ongoing practice.

In contrast to many intervention studies, the present study resembled a real-life situation of primary schools in Southwest Uganda where probiotic yogurt was already served. In order to allow baseline measurement for this study, ten new schools were recruited to start with the yogurt or milk school feeding program. During the study there was minimal distortion of daily practices at the participating schools. In addition, no dietary or behavior guidelines were given to the participating children and their parents, except for the daily consumption of 100 ml of probiotic yogurt or milk.

The comparative interrupted time series included a relatively high frequency (almost daily) of data collection over the course of 10 weeks. Compared to most nutritional intervention studies, where data is only collected at beginning, end, and sometimes mid-point, the CITS allows for a more precise observation of trends during baseline and the consumption phase of milk or yogurt, such as the occurrence of cross infections between children during the first weeks after the beginning of the school term.

The yogurt was locally made by producers who were not only recruited to produce the product for this study, but who were already supplying the probiotic yogurt to neighboring schools. The producers received both technical as well as business guidance on a periodic basis, and the quality of the milk and probiotic yogurt products was regularly tested.

In terms of health benefits, the children enrolled in the yogurt school feeding program profit from the multiple benefits from a functional fermented food (37). More specifically, in this yogurt the probiotic *L. rhamnosus* yoba 2012 has propagated in the milk matrix during the fermentation. As a consequence, and contrary to concentrated probiotic dietary supplements, the fermented milk matrix also contains bioactive metabolites like acids and vitamins produced by *L. rhamnosus* and *S. thermophilus* bacteria during milk fermentation.

### Limitations of This Study

Although there is a large body of literature pointing to the health benefits of *L. rhamnosus* yoba 2012 (*L. rhamnosus* GG), currently no studies are available on the health benefits of *S. thermophilus* C106. As a consequence, for all results presented above, we cannot exclude the role of *S. thermophilus* C106, or synergistic effects between the two bacteria.

The study was non-randomized, and participation in either the yogurt or milk group depended on the school's and parents' choice to either enroll in the school yogurt or in the school milk program. As the probiotic yogurt program was slightly more expensive, and as yogurt is an unknown product to part of the rural population, the yogurt program was particularly popular among schools where children from higher economic status attended.

This study aimed to investigate the impact of probiotics present in the locally produced probiotic yogurt on the lives of children in Southwest Uganda as part of their regular diet. Hence, children were not advised to change their normal dietary habits. Rather, their dietary habits have been assessed, and used as a parameter for calculation of propensity scores of the children (see section Methods). More specifically, different schools received different school meals in addition to the 100 ml of milk or yogurt. Furthermore, the high level of milk consumption and significant level of consumption of fermented foods of children at baseline and during the subsequent yogurt or milk consumption period, may have reduced the additional impact of the probiotic yogurt. The health benefits studied here may have partially resulted from the consumption of fermented foods during the study period (37).

A challenge related to the duration of the study was set to a school term of 12 weeks. In Uganda, school fees are paid by the parents every school term, and usually parents wait till the last moment to start collecting the fees, which need to be paid in order to get access to the school. Hence, at the beginning of the school term, absenteeism is very high. Furthermore, toward the end of the term, absenteeism again increases, as children leave school early for holidays. Consequently, due to high absence of children in the first week of the study and early start of the holidays in 8 of the 10 schools in the last (twelfth) week of the study, these weeks were excluded from data analysis.

In addition, many schools had put restrictions on the time and duration of the daily visitations of the school nurse. Hence, the nurses were sometimes rushed, which may have caused overlooking of certain incidences of infections or symptoms. This may have contributed to an irregularity in reports of skin conditions or common cold symptoms. For instance, sometimes skin infection symptoms were not continuously indicated, but on separate days within a period of e.g., 10 days. However, in view of the normal development of skin infections (38, 39), it is unlikely that the condition would disappear 1 day and would return the following day. The gaps in reporting could also have been caused by the child being absent, the nurse not attending the school on that particular day, or the hair growth of the child. (Long hair makes the skin condition harder to detect, and children usually go for shaving every 1 or 2 weeks, after which the condition of the head skin becomes much more visible).

In case of missing data on the reporting of skin infections, interpolation of the data points was done before calculating the total incidence of infections. For the subsequent data analysis, it was assumed that gaps smaller than 10 days actually represented one ongoing episode of skin infection. For absence of skin infection indications of more than 10 days, we assumed it concerned two different episodes of skin infection. From all the possible data points that could have been reported, 82% were actually collected. Furthermore, at schools Faith Memorial, Jireh Junior (milk group) and BDA junior (yogurt group) it was noted that the responsible nurse had not recorded occurrences of skin infections at all (Supplementary Figures 1A,B). In order to avoid a bias in the data interpretation, the authors decided to exclude these schools in analysis of skin infection symptoms and the number of evaluated children has been adjusted in the corresponding Tables and Figures.

In case of missing data for common cold symptoms, gaps of data for every individual school were closed by linear interpolation of the incidence of common cold on days adjacent to the gap. Next, the average incidence of common cold in either the yogurt or the milk group was calculated. From all the possible data points that could have been reported, 85% of the data was available, while the remaining 15% was based on interpolation.

### Skin Conditions

The incidence rate of *tinea capitis* was in the same range as reported by other authors who conducted studies among African children (5, 19, 20). The increase in skin conditions during the first 6 weeks of the study may be due to the fact that most of these skin conditions are highly transmittable (39), and hence their prevalence increases when children come back to school after holidays. The difference between incidence of skin conditions at baseline in the milk and yogurt group is also similar to differences observed by other authors who reported highly varying incidence rates of tinea capitis between schools in the same region (19). Differences in baseline values between study groups are common in non-randomized studies, but compromise the robustness. The decreasing incidence of skin infections in the yogurt group relative to the milk group at the start of the consumption period (Figure 2B), suggests a positive effect of the consumption of probiotic yogurt and corresponds to observations made during a previous pilot study we conducted in Uganda among 245 children (24). With regard to atopic dermatitis, from a review of 13 studies on the incidence of this skin disease among children below 3 years of age, we conclude there is a preventive as well as reducing effect by *L. rhamnosus* GG (40). Also, other studies showed that intake of the probiotic *L. rhamnosus* GG led to reduction of eczema and allergic reactions (15, 41, 42). However, in the current study the overall incidence of atopic dermatitis and eczema was too low, and there may be too many confounding factors in order to draw conclusions about effects of probiotic yogurt on these specific conditions.

Notwithstanding the positive results observed for the probiotic yogurt group vs. the milk group, we would like to refrain from making definitive conclusions, and instead propose follow-up studies with an adjusted study design. Especially, the differences at baseline, the increase of infection at the start of the consumption in both groups, as well as a number of possible confounding factors, including differences in individual diet, behavior and social-economic situation of the participating children and their families, has led to our recommendation to conduct follow-up studies over a longer period of time, in a more uniform background situation, and with additional controls such as a study group without yogurt or milk.

### Common Cold

The increase of common cold during the baseline in both control and yogurt group may be explained by its highly infectious nature among children in the classroom. Also, from other studies we know that the risk ratio is 1.9 of contracting common cold for a young child attending day-care, compared to a child kept at home (43). The slightly decreasing incidence of common cold in the yogurt group relative to the milk group during the consumption period (Figure 3B) only indicates a trend., It should be noted that previous studies and meta-analyses with different study designs showed more pronounced effects of *L. rhamnosus* GG on different types of respiratory tract infections, possibly through direct antimicrobial effects, improved mucosal barrier function and immunomodulating activity (14, 44–51), see also Supplementary Table 1. The reduction of common cold symptoms after the start of the consumption of milk and yogurt in both groups may be explained by seasonal influences or by an immune-boosting effect of consumption of milk and yogurt, e.g, as a result of its nutritional value of micro- and macro nutrients. Previous research has shown that milk contains components including fatty acids, lauric acid and zinc that have antiviral properties or play a role in immune function (52), as well as bioactive peptides (53–55). As reported above for skin diseases, also for the incidences of common cold, we would like to refrain from making definitive conclusions, and we rather propose follow-up studies with an improved study design as summarized above.

### Absenteeism

An increase in school attendance as a result of school feeding programs has been reported by several other authors. Studies among low-income or food-insecure children in North-Uganda (56), Ghana (57), and South-Ethiopia (58) showed increases in school attendance of 13 percent points, 15 and 13%, respectively, as a result of offering meals at school. A study among children in Kenya with a baseline attendance of 95%, still showed an increase in attendance of 1–4 percentage points for different school meals provided (59). In the present CIRS, the observed school attendance was already relatively high and all schools had already implemented school feeding programs, in addition to the supplementation of milk or yogurt. Consequently, we assume that the measured effect of the provision of dairy products on school attendance was almost negligible.

### Anthropometric Indicators

The anthropometric indicator WAZ slightly decreased during the study. As a result, in both groups we also observed during the study period a reduction in BAZ value. We hypothesize that children eat less or use more energy when they are at school compared to when they are at home during the holidays. This observation argues for additional school feeding programs with preferably higher quantities of food than the 100 ml of daily milk or yogurt servings provided in the present study.

A study with school milk in Iran did not find significant changes in anthropometric indicators for boys after 3 months of intervention (60). Only one study in New Guinea found more growth in children supplemented with milk compared to the control group. However, the sample size in this study was extremely small (61). A study in Kenya running for a period of 2 years indicated improved growth in children below 6 years of age, and in stunted children (62).

A retrospective study showed a correlation between higher milk consumption and higher values for BMI and height for 4 year old children (63). Similarly, a retrospective cohort study of 12,376 children in South East Asia indicated significant lower levels of stunting, underweight, vitamin A and vitamin D deficiency for children who would consume dairy on a daily basis, compared to those who did not consume dairy products (64).

From earlier research with *L. rhamnosus* GG it is known that this strain can improve feeding tolerance and nutrient absorption (increased proliferation of villus cells) (65, 66), and could contribute to increased weight gain in children. Moreover, *L. rhamnosus* GG has shown to bind aflatoxin B1, thereby reducing its absorption in the intestine, hence reducing aflatoxin-associated pathogenicity including stunting (67–70). In view of the observations made in our study, we hypothesize that a measurable positive effect as a result of milk and probiotic yogurt consumption is likely to be found after a longer time period of consistent daily consumption of milk or yogurt. Since the present study did not include a group which did not consume a dairy product, we can only compare the impact of milk vs. probiotic yogurt.

### Implications

In this study, we compared the consumption of probiotic yogurt with the consumption of plain milk in a real-life school setting. This means that besides the milk and the yogurt the children also consumed their regular maize and beans dominated diet, as well as occasional consumption of dairy products and fermented foods. Since in Southwest Uganda the majority of the schools do not serve any dairy product, and a majority of children suffer from malnutrition, we think it would be useful to conduct a study comparing not only the effects of probiotic yogurt vs. milk, but also vs. a control group that does not receive any dairy product. In such a study set-up it should be able to identify the cumulative benefits of both the nutritious components of dairy products, the bioactives produced during fermentation, as well as probiotic bacteria.

Referring to the 20,000 children that already participate in the probiotic yogurt school feeding program, we can conclude that consumption of locally produced probiotic yogurt among pre-primary school children in Southwest Uganda has gained broad acceptance. The program which started in 2018 is growing and gaining further popularity among school management committees, parents, pupils and producers in the region. It clearly provides economic opportunities through employment creation for producers, a market for milk of local farmers, and value addition to local products (24, 25). In addition, schools that participate in the program testify to have become more attractive for pupils and parents compared to schools which do not offer such nutritious and tasty dairy products to their pupils.

## Conclusion

In this comparative interrupted times series at pre-primary schools in Southwest Uganda, we evaluated the effect of the consumption of probiotic yogurt vs. milk on the incidence of skin infections, common cold, absenteeism and anthropometric parameters among 1,116 children. Following a base-line measurement period, the pre-primary school children received probiotic yogurt or milk for a period of 8 weeks. The consumption of probiotic yogurt as conducted in this real life school feeding program showed a reducing effect on the incidence of skin infections compared to consumption of milk (*p* < 0.0001), and a reducing trend on common cold symptoms (*p* = 0.09). Anthropometric indicators and level of absenteeism did not show significant differences between yogurt and milk groups during the study period.

Since this study was done in a real-life setting, effects of confounding factors cannot be excluded. In addition, heterogeneity between control and yogurt group was observed during the 3 weeks of baseline period. Furthermore, we noticed a strong degree of cross-infection for skin infection related symptoms and common cold from the start of the study and during part of the dairy consumption period causing fluctuations in the occurrence of skin infection and common cold related incidences over time. Notwithstanding this observed positive effect and trend of probiotic yogurt on the incidences of skin infections and common cold, respectively, we consider the results of this comparative interrupted time series inconclusive. Before drawing definitive conclusions on the health impact of probiotic yogurt consumption through school feeding programs, follow-up studies are required over a longer period of time, with additional controls and a more uniform background situation for probiotic yogurt and milk groups.

## Data Availability Statement

The original contributions presented in the study are included in the article/Supplementary Materials, further inquiries can be directed to the corresponding author/s.

## Ethics Statement

The studies involving human participants were reviewed and approved by Mbarara University of Science & Technology Research Ethics Committee. Written informed consent to participate in this study was provided by the participants' legal guardian/next of kin.

## Author Contributions

NW, AW, AN, RK, and WS: conceptualization. NW, RK, and WS: methodology. NW, AW, and AN: investigation. NW: writing–original draft preparation. RK and WS: writing–review and editing. All authors: contributed to the article and approved the submitted version.

## Conflict of Interest

The Yoba for Life foundation distributes and sells ready-to-use sachets with dried bacterial starter cultures at cost price, through a network of partners and volunteers to facilitate the local production of dairy and cereal-based products by controlled bacterial fermentation. African fermented products made with the Yoba starter culture, are not marketed by the foundation as such, but the Yoba for Life foundation stimulates local production and ownership, allowing income-generating activities for African small-scale entrepreneurs in the food sector. RK and WS are co-founders of the Yoba for Life foundation (2009), a non-profit organization, accredited by the Dutch Tax Authorities as a Public Benevolent Institution (PBI), which aims to promote local production and consumption of fermented products in Africa. NW is the Country Coordinator of the Yoba for Life Foundation in Uganda. In name of the Yoba for Life Foundation AN and AW provided technical support to local probiotic yogurt production units.
