# Supplementary material for: A Comparative Interrupted Times Series on the Health Impact of Probiotic Yogurt Consumption Among School Children From Three to Six Years Old in Southwest Uganda
Source: Front Nutr. 2020 Dec 9;7:574792. doi: 10.3389/fnut.2020.574792 (PMC7756026; doi:10.3389/fnut.2020.574792)
Supplement: Supplementary file 1 [file Data_Sheet_1.DOCX]

SUPPLEMENTARY MATERIAL

**Supplementary Table 1**: Summary of studies investigating the effect of *Lactobacillus rhamnosus* GG (LGG) on respiratory tract infections (RTI) in day care centres.

| **Place and design** | **Subjects** | **Intervention** | **Outcomes** | **Ref.** |
| --- | --- | --- | --- | --- |
| **Finland, randomized, double-blind placebo-controlled trial** | 517 children, 1-6 years old | Milk (placebo) or milk with LGG, 260 ml/day, 7 days per week, 7 months | Relative reduction of 17% in the  number of children suffering from RTI with complications and lower respiratory tract infections (Odds Ratio OR 0.75, 95% CI 0.52 to 1.09) | [20] |
| **Croatia, randomized, double-blind placebo-controlled trial** | 742 hospitalized children, average age 10 years | Fermented milk (placebo) or yoghurt containing LGG, 100 ml/day, average 4-5 days (duration of hospitalization). | Significantly reduced risk of RTI in the LGG group compared to the placebo group (Relative Risk RR: 0.38 [95% CI: 0.18–0.85]; | [21] |
| **Croatia, randomized, double-blind placebo-controlled trial** | 281 children, 1-7 years old | Fermented milk (placebo) or yoghurt containing LGG, 100 ml/day, 7 days per week, 3 months | The risk of RTI significantly reduced in the LGG group compared with the placebo group (RR 0.63, 95% CI 0.51 to 0.79). | [22] |
| **Argentina, Randomized controlled trial** | 298 children, 2-5 years old | Regular yoghurt (placebo) or yoghurt containing *L. rhamnosus* CRL 1505, 100 ml/day, 5 days per week, 6 months | The incidence of RTI in the intervention group was 31%, versus 69% in the control group. The study found no differences in anthropometric indicators between the intervention and control group. | [23] |
| **Finland, randomized, double-blind, placebo-controlled trial** | 523 children, 2-6 years old | Milk (placebo) or milk containing LGG, 400 ml/day, 7 days per week, 28 weeks | Insignificant reduction in the sick days due to RTI in the intervention group. Incidence rate ratio (IRR) 0.97; 95% CI:0.94–1.00; P=0.098) | [24] |
| **Finland, randomized, double-blind, placebo-controlled trial** | children in the intervention group with faecal LGG recovery (*n* = 81), and children in the control group with no faecal LGG recovery (*n* = 47), 2-6 years old | Milk (placebo) or milk containing LGG, 400 ml/day, 7 days per week, 28 weeks | IRR of RTI in intervention group 0.83; 95% CI: 0.78–0.88; P<0.001. | [24] |

Note: Studies with different probiotic strains or with a combination of strains [18, 19] have not been included in this table.

**Supplementary Picture 1:** Example of a 100 ml pack of locally produced probiotic yoghurt


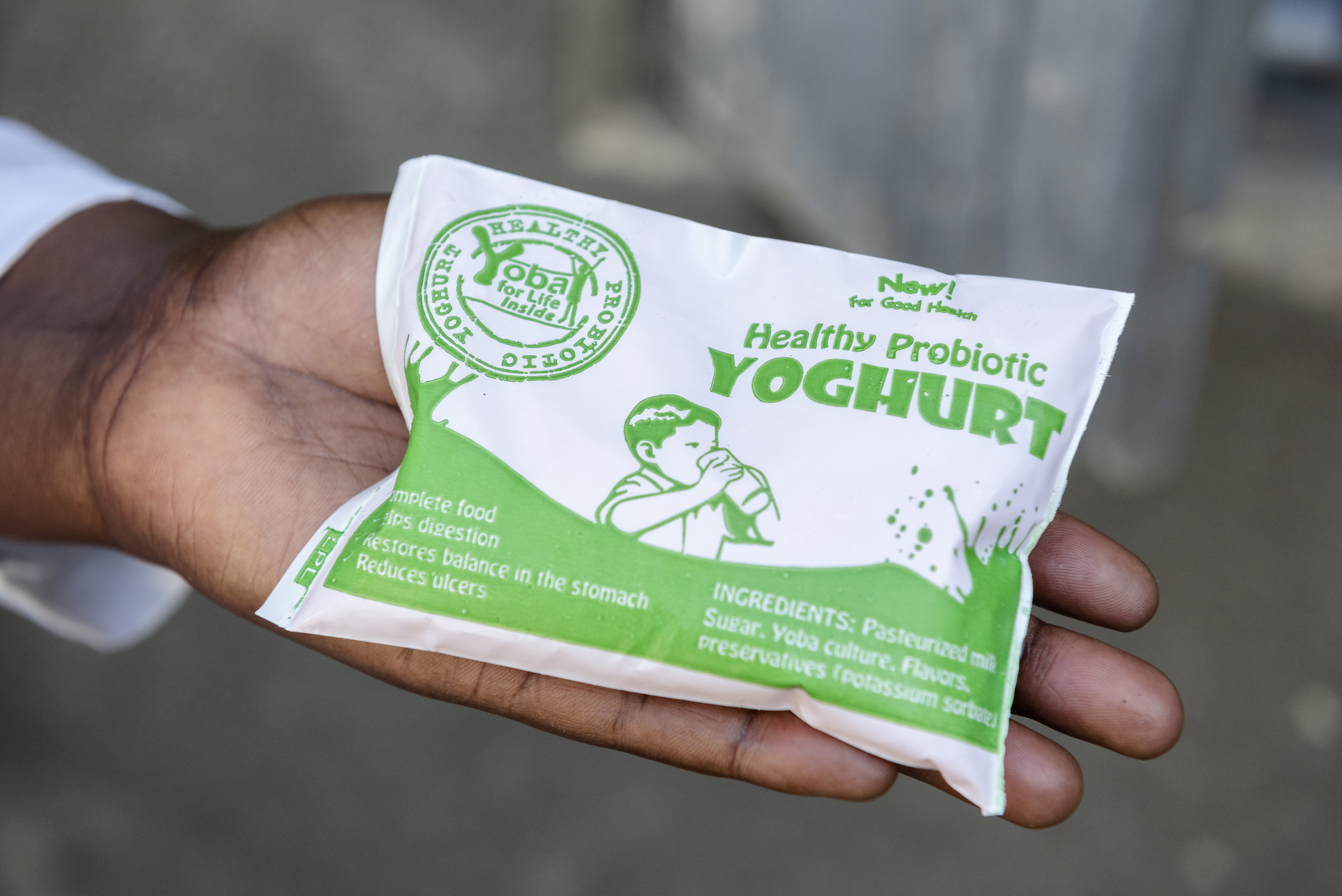


**Supplementary Figure 1A**: Incidence of skin diseases per school in the milk group .

**Supplementary Figure 1B**: Absolute incidence of skin diseases per school in the yoghurt group**.**

**Supplementary Figure 1C**: Incidence of skin diseases in the yoghurt group (open circles) and the milk group (closed circles).

**Supplementary Figure 1D**: Incidence of common cold per school in the milk group.

**Supplementary Figure 1E**: Incidence of common cold per school in the yoghurt group.

**Supplementary Figure 1F**: Absolute incidence of common cold in the yoghurt group (open circles) and the milk group (closed circles).

**Supplementary Table 2:** Parameter estimates, standard errors and *P*-values for performed regressions

|  |  | Coefficient | Standard error | *t*-statistic | *P*-value |
| --- | --- | --- | --- | --- | --- |
| Common cold | Intercept | 26 | 15 | 1.8 | 0.088 |
|  | X Variable | -0.00059 | 0.00034 | -1.8 | 0.088 |
| Rhinitis | Intercept | 0.017 | 0.020 | 0.85 | 0.40 |
|  | X Variable | -0.00017 | 0.00040 | -0.41 | 0.68 |
| Cough | Intercept | -0.045 | 0.0084 | -5.4 | 5.6E-06 |
|  | X Variable | 0.00027 | 0.00017 | 1.6 | 0.13 |
| Skin | Intercept | 78 | 11 | 7.3 | 1.2E-08 |
|  | X Variable | -0.0018 | 0.00025 | -7.3 | 1.2E-08 |

# Supplementary Figure 2A: The actual and model-fitted SMR-weighted incidence of rhinitis infections. Data were collected in the yoghurt group (*n* = 584) group and milk group  (*n* = 532) between day 7 and day 73, expressed as a percentage of the total children under observation. Δ = data points yoghurt group; solid line = trend yoghurt group; ● Data points milk group; dotted line = trend milk group. The vertical dotted line indicates the transition from baseline (day 1-21) to yoghurt/milk consumption period (day 22-84).

#

**Supplementary Figure 2B: The actual and model-fitted SMR-weighted incidence of cough infections.** Data were collected in the yoghurt group (n = 584) group and milk group (n = 532) between day 7 and day 73, expressed as a percentage of the total children under observation. Δ = data points yoghurt group; solid line = trend yoghurt group; ● Data points milk group; dotted line = trend milk group. The vertical dotted line indicates the transition from baseline (day 1-21) to yoghurt/milk consumption period (day 22-84).

**Supplementary Table 3:** Anthropometric parameters.

|  | **Average age (months)** | **Average weight (kg)** | **Average height (cm)** | **HAZ** | **WAZ** | **BAZ** |
| --- | --- | --- | --- | --- | --- | --- |
| **Yoghurt** |  |  |  |  |  |  |
| Week 1 | 57.6 (± 13.6) | 17.8 (± 3.0) | 105.9 (± 8.4) | -0.41 | -0.07 | 0.30 |
| Week 3 | 58.0 (± 13.6) | 17.7 (± 2.9) | 106.2 (± 8.3) | -0.39 | -0.14 | 0.17 |
| Week 7 | 58.7 (± 13.6) | 17.9 (± 3.0) | 106.7 (± 8.3) | -0.37 | -0.13 | 0.17 |
| Week 11 | 59.7 (± 13.6) | 17.9 (± 3.0) | 107.0 (± 8.3) | -0.41 | -0.19 | 0.13 |
| **Milk** |  |  |  |  |  |  |
| Week 1 | 58.6 (± 16.6) | 18.1 (± 3.3) | 107.3 (± 10.1) | -0.20 | -0.01 | 0.17 |
| Week 3 | 59.2 (± 16.6) | 18.2 (± 3.4) | 107.6 (± 10.0) | -0.23 | -0.03 | 0.16 |
| Week 7 | 59.9 (± 16.6) | 18.3 (± 3.4) | 108.1 (± 10.1) | -0.23 | -0.09 | 0.08 |
| Week 11 | 60.8 (± 16.6) | 18.4 (± 3.4) | 108.4 (± 10.0) | -0.24 | -0.08 | 0.10 |

Average age, weight, height and anthropometric indicators height-for-age (HAZ), weight-for-age (WAZ) and Body Mass Index-for-age (BAZ) at the four different measurement points during the study for the intervention (yoghurt, *n* = 424) and control (milk, *n* = 379) group. Only children from whom all 4 measurement points were taken and the date of birth was known, were included in this analysis. Measures are assessed in reference to the WHO standard growth curves, and are expressed as Z-scores (see methods section). A HAZ value of ≤-2 indicates stunted growth, a WAZ value of ≤-2 indicates wasting, and a BAZ of ≤-2 indicates underweight.

**Supplementary Table 4:** *P*-values for inter- and intra-group differences of collected anthropometric parameters during baseline and endline..

|  |  | **Yoghurt end line** | **Milk baseline** |
| --- | --- | --- | --- |
| **HAZ** | Yoghurt baseline | 0.98 | 0.039 |
|  | Milk endline | 0.058 | 0.025 |
| **WAZ** | Yoghurt baseline | 0.00007 | 0.34 |
|  | Milk endline | 0.054 | 0.010 |
| **BAZ** | Yoghurt baseline | < 0.00001 | 0.092 |
|  | Milk endline | 0.57 | 0.080 |

# Supplementary Text 1: Content of mobile application forms

## Form 1: Incidence of diseases form

1. Name of child (from drop-down list)
2. Date
3. Disease
   1. Common cold
      1. Mild cough
      2. Severe cough
      3. Severe runny nose
      4. Mild runny nose
      5. Blocked nose
      6. Severe sore throat
      7. Mild sore throat
      8. Fever
      9. Headache
      10. Malaise
      11. Loss of appetite
   2. Throat infection
      1. Sore throat
      2. Fever
      3. Red and swollen tonsils
      4. White patches or streaks of pus on tonsils
      5. Tine red spots on the area at the back of the roof of the mouth
      6. Headache
      7. Nausea
      8. Vomiting
      9. Swollen lymph nodes in the neck
      10. Body aches
      11. Body rash
   3. Pneumonia
      1. Mild cough
      2. Severe cough
      3. Fever
      4. Fast breathing
      5. Chest moves in or retracts during inhalation
   4. Bronchitis
      1. A history of productive cough (producing mucus) on most days for at least 3 months
   5. Ear Infection
      1. Collection of fluid in the ear
      2. Mild hearing loss
      3. Severe hearing loss
   6. Skin disease
      1. Specify
         1. Pyoderma/Bacterial Skin infection
            1. Impetigo
            2. Ecthyma
            3. Impetigo contagiosa
            4. Furuncle
            5. Carbuncle
            6. Tropical ulcer
            7. Folliculitis
            8. Others - Specify
            9. Unknown
         2. Scabies
         3. Ectoparasitoses
         4. Tinea Capitis
         5. Superficial mycoses
         6. Benign viral tumours
         7. Dermatitis
         8. Unknown
      2. Extend of the coverage
         1. One part of the body
         2. Several body parts affected
         3. Full body affected
      3. Severity of the skin rash
         1. Mild
         2. Moderate
         3. Severe
      4. Take a picture
   7. Diarrhoea
      1. Passage of 3 loose stools per day
      2. Passage of 4 or more loose stools per day
   8. Malaria
      1. Fever
      2. Headache
      3. Chills
      4. Pains in the joints
   9. Others – Specify
4. Does the child take medicine?
   1. Antibiotics – Specify
   2. Painkillers - Specify
   3. Other – Specify

## Form II: Parent Interview Form

1. Interview period
   1. Beginning of term
   2. Mid-term
   3. End of Term
2. Date
3. Name of parent
4. Contact of parent
5. Sex of household head
   1. Female
   2. Male
6. Age of household head
7. Number of people in household in the different categories
   1. Females < 5 years
   2. Males < 5 years
   3. Females 6 – 13 years
   4. Males 6-13 years
   5. Females 14 – 59 years
   6. Males 14 – 59 years
   7. Females > 60 years
   8. Males > 60 years
8. Level of education of household head
   1. No formal education
   2. Primary
   3. Secondary
   4. Tertiary
9. Does your house have a toilet facility?
   1. Yes
   2. No
10. Source of drinking water
    1. Protected well or spring
    2. Borehole
    3. Open spring or well
    4. Surface water
    5. Rain Water
    6. Piped water
11. Water treatment for drinking water
    1. Boil
    2. Add bleach or chlorine
    3. Strain with cloth
    4. Let it stand and settle
    5. Other
12. Name of child (from drop-down list)
13. HIV status of child
    1. Positive
    2. Negative
    3. Non-disclosed
14. Does the child have allergy or asthma?
    1. Yes
       1. Lactose intolerance
       2. Asthma
       3. Cold weather
       4. Itching eyes
       5. Skin problem
       6. Others - Specify
    2. No
15. Diseases the child has suffered from in the last month / since the last interview
    1. Ear infection
    2. Throat infection
    3. Common cold
    4. Pneumonia
    5. Bronchitis
    6. Diarrhoea
    7. Skin infections
    8. Malaria
    9. Others
    10. Unknown
    11. None
16. Has your child missed school due to sickness in the last month / since the last interview?
    1. Yes
    2. No
17. Did your child use medication in the last month / since the last interview?
    1. Yes
       1. Antibiotics
          1. Septrin
          2. Amoxicillin
          3. Erythromycin
          4. Metronidazole
          5. Ampiclox
          6. Azithromycin
          7. Tetracycline
          8. Ceftriaxone
          9. Seomycin/cyclocerine
          10. Ciprofloxacin
          11. Others - specify
       2. Painkillers
          1. Panadol / Paracetamol
          2. Aspirin
          3. Ibuprofen
          4. Diclofenac
          5. Others - Specify
       3. Malaria medicine
       4. Eye drops
       5. Herbal medicine
       6. Cough syrup
       7. Skin ointment
       8. Cough or flue tablets
       9. Piriton or allergy tablets
       10. Other - Specify
    2. No
18. How long ago did you last de-worm your child?
    1. Less than 3 months ago
    2. More than 3 months ago

*Question 12 to 18 are repeated if the parent has more children*

|  | Estimated consumption frequency per week | | | | Eaten in the past 24 hours? |
| --- | --- | --- | --- | --- | --- |
|  | Once per week | Twice per week | Daily | Less than once per week |  |
| Cereals and grains |  |  |  |  |  |
| Fermented cereals and grains |  |  |  |  |  |
| Roots and tubers |  |  |  |  |  |
| Pulses and nuts |  |  |  |  |  |
| Vegetables |  |  |  |  |  |
| Fruits |  |  |  |  |  |
| Meat |  |  |  |  |  |
| Fish |  |  |  |  |  |
| Eggs |  |  |  |  |  |
| Milk and other dairy products |  |  |  |  |  |
| Fermented milk |  |  |  |  |  |
| Oil/fat/butter |  |  |  |  |  |
| Sugar or sweets |  |  |  |  |  |
| Condiments or spices |  |  |  |  |  |
